# Assessment of the impact of mitochondrial genotype upon drug-induced mitochondrial dysfunction in platelets derived from healthy volunteers

**DOI:** 10.1007/s00204-021-02988-3

**Published:** 2021-02-13

**Authors:** Amy L. Ball, Katarzyna M. Bloch, Lucille Rainbow, Xuan Liu, John Kenny, Jonathan J. Lyon, Richard Gregory, Ana Alfirevic, Amy E. Chadwick

**Affiliations:** 1 Department of Pharmacology and Therapeutics, MRC Centre for Drug Safety Science, University of Liverpool, Liverpool, UK; 2 The Wolfson Centre for Personalised Medicine, Department of Pharmacology and Therapeutics, University of Liverpool, Liverpool, UK; 3 Centre for Genomic Research, Institute of Integrative Biology, University of Liverpool, Liverpool, UK; 4 GSK GlaxoSmithKline, Safety Assessment, Ware, UK

**Keywords:** 2-Hydroxyflutamide, Adverse drug reaction, Flutamide, Haplogroup, Mitochondria, mtDNA, Platelets, Tolcapone

## Abstract

Mitochondrial DNA (mtDNA) is highly polymorphic and encodes 13 proteins which are critical to the production of ATP via oxidative phosphorylation. As mtDNA is maternally inherited and undergoes negligible recombination, acquired mutations have subdivided the human population into several discrete haplogroups. Mitochondrial haplogroup has been found to significantly alter mitochondrial function and impact susceptibility to adverse drug reactions. Despite these findings, there are currently limited models to assess the effect of mtDNA variation upon susceptibility to adverse drug reactions. Platelets offer a potential personalised model of this variation, as their anucleate nature offers a source of mtDNA without interference from the nuclear genome. This study, therefore, aimed to determine the effect of mtDNA variation upon mitochondrial function and drug-induced mitochondrial dysfunction in a platelet model. The mtDNA haplogroup of 383 healthy volunteers was determined using next-generation mtDNA sequencing (Illumina MiSeq). Subsequently, 30 of these volunteers from mitochondrial haplogroups *H*, *J*, *T* and *U* were recalled to donate fresh, whole blood from which platelets were isolated. Platelet mitochondrial function was tested at basal state and upon treatment with compounds associated with both mitochondrial dysfunction and adverse drug reactions, flutamide, 2-hydroxyflutamide and tolcapone (10–250 μM) using extracellular flux analysis. This study has demonstrated that freshly-isolated platelets are a practical, primary cell model, which is amenable to the study of drug-induced mitochondrial dysfunction. Specifically, platelets from donors of haplogroup *J* have been found to have increased susceptibility to the inhibition of complex I-driven respiration by 2-hydroxyflutamide. At a time when individual susceptibility to adverse drug reactions is not fully understood, this study provides evidence that inter-individual variation in mitochondrial genotype could be a factor in determining sensitivity to mitochondrial toxicants associated with costly adverse drug reactions.

**Supplementary Information:**

The online version contains supplementary material available at 10.1007/s00204-021-02988-3.

## Introduction

Drug-induced mitochondrial dysfunction is a major pathway associated with adverse drug reactions which has led to the withdrawal of therapeutic compounds from the market (Massart et al. 2018). Nonetheless, many of the compounds that induce mitochondrial dysfunction can be administered to the vast majority of patients without reaction, indicating the presence of patient-specific risk factors (Chu et al. 1998; Olanow and Watkins 2007).

The mitochondrial genome is a 16,569 base-pair circular DNA encoding 22 tRNAs and 2 rRNAs as well as 13 subunits of the electron transport chain (ETC) respiratory complexes and ATP synthase, together constituting the necessary machinery for oxidative phosphorylation. In contrast to the nuclear genome, the mitochondrial genome is maternally inherited, resulting in negligible recombination; thus dividing the population into discrete haplogroups characterised by a set of single nucleotide polymorphisms (SNPs) (Taylor and Turnbull 2005).

Several studies have indicated that the mitochondrial genome may be a factor in the onset of adverse drug reactions, including those associated with linezolid and streptomycin (Chen et al. 2007; Pacheu-Grau et al. 2012; Penman et al. 2020). In addition, drug-induced mitochondrial dysfunction is an important part of preclinical screening in the pharmaceutical industry. However, research to elucidate the triad of the mitochondrial genome, drug-induced mitochondrial dysfunction and adverse drug reactions is limited. This is due, in-part, to current screening tools which neglect the inter-individual genetic diversity present at the population level (Will and Dykens 2014). Thus, research is required to define the potential importance of mitochondrial genetics in defining individual susceptibility to adverse drug reactions, as well as to provide the practical models required for the field to advance. Therefore, the aim of this study was to evaluate differences in drug-induced mitochondrial dysfunction between mitochondrial haplogroups using freshly isolated human platelets; isolated from healthy volunteers of known haplogroup. Platelets were selected due to their accessibility via a minimally invasive technique (venepuncture) and their abundance of mitochondria and mitochondrial DNA (mtDNA). The anucleate nature of platelets also enables the effect of mtDNA variation to be determined without interference from the nuclear genome (Thon and Italiano 2012).

The present study examined the mitochondrial genome of a cohort of 383 healthy volunteers from within an existing DNA biobank using whole genome sequencing to determine mitochondrial haplogroup. Following haplogroup assessment, volunteers from mitochondrial haplogroups *H*, *J*, *T* and *U* (the four most common haplogroups in the cohort and in England) were recalled to donate fresh whole blood from which platelets were isolated (Eupedia 2016). Basal bioenergetic function was assessed using extracellular flux (XF) analysis to quantify key parameters of mitochondrial respiratory function and individual respiratory complex-driven respiration.

The effect of haplogroup upon susceptibility to drug-induced mitochondrial dysfunction was then examined using three therapeutic compounds that are known to induce mitochondrial dysfunction via two classic modes of direct ETC damage: flutamide and 2-hydroxyflutamide (primary metabolite of flutamide) via respiratory complex inhibition, and tolcapone via an uncoupling mechanism. All three compounds are associated with adverse drug reactions and have either been withdrawn or received a boxed warning as a result (Chu et al. 1998; Olanow and Watkins 2007; Ball et al. 2016; Longo et al. 2016; Grünig et al. 2017). Compared with its parent compound, 2-hydroxyflutamide has a higher C_max_ (4.4 µM vs. 72.2 nM) and is an inhibitor of respiratory complex II as well as respiratory complex I; therefore, the metabolite was selected to investigate the association of haplogroup upon susceptibility to perturbations at specific respiratory complexes (Ball et al. 2016). Finally, the results from basal and drug-induced mitochondrial (dys)function were pooled from platelets of all haplogroups to assess the utility of measuring basal mitochondrial function in platelets as a means to predict susceptibility to drug-induced mitochondrial dysfunction.

## Materials and methods

### Materials

All DNA library preparation reagents were purchased from Fluidigm (CA, USA) unless otherwise stated. All sequencing reagents were purchased from Illumina (CA,USA) unless otherwise stated. All XF assay consumables were purchased from Agilent Technologies (CA, USA) unless otherwise stated. All other reagents and chemicals were purchased from Sigma Aldrich (Dorset, UK) unless otherwise stated.

### Cohort

This study was approved by the North West of England Research Ethics Committee and all participants gave written informed consent. Study eligibility and exclusion criteria have been published previously (Alfirevic et al. 2012).

Samples were selected from a study by Faulkner et al. in which 600 healthy volunteers were recruited from the North West of England and 100 mL of blood was collected for DNA extraction (Faulkner et al. 2016). The most recently collected 384 DNA samples were selected for mitochondrial genotyping to maximise volunteer recall. Volunteer confidentiality was maintained at all times by double coding DNA samples and by restricting access to participant personal data to trained clinical personnel.

Volunteers were 64% female and 36% male with a mean age of 29 years (range 18–60 years). The ethnicity of the volunteers also reflected regional diversity; primarily Caucasian (white north European ancestry; 84%) with Asian Indian (6%), Chinese (4%) and Black (1%) as minority populations.

### DNA isolation and library preparation

Multiplex amplicon tagging using the Fluidigm 48.48 Access Array Integrated Fluidics Circuit (IFC) System was performed on 384 DNA samples according to the manufacturer’s protocol using primers designed for the mitochondrial genome (Fluidigm Assay Design Report 5758_AAP_15.D1; cycling conditions detailed in Supplementary Fig. 1). Sample input was 100 ng and all harvested PCR products were amplified and tagged with a unique barcode (1–384; conditions for incorporation of Illumina barcodes are detailed in Supplementary Fig. 2).

All barcoded, amplified PCR products generated on the 48.48 Access Array IFC underwent quality control using the Fragment Analyser and concentrations were measured by a Qubit HS kit using a 96-well plate reader. The PCR products were then pooled to create four PCR product libraries using the Mosquito X1 (TTP labtech, Melbourn, England). Following pooling, the libraries were cleaned using Ampure XP beads (1:1). The quantity and quality of each pool was assessed by the Bioanalyser (Agilent 2000) and subsequently by qPCR using the Illumina Library Quantification Kit (Kapa Biosystems, UK) on a Light Cycler LC480II (Roche, Switzerland) according to the manufacturer’s instructions. The four PCR product libraries were pooled in an equimolar ratio prior to sequencing.

### MtDNA sequencing

Template DNA (from PCR product libraries) was denatured according to the protocol described in the Illumina cBot user guide. Briefly, 5 µL of non-denatured library was added to 5 µL 0.1 M NaOH and 5 µL 200 mM Tris-HCl pH 8.0. The library mix (10 pM) and Read1, Read2 and Read3 sequencing primers (diluted with hybridisation buffer) were then added to the MiSeq reagent cartridge, pre-loaded with clustering and sequencing reagents. The 2 $\times$ 150 bp paired-end sequencing was performed on one lane of an Illumina MiSeq with v2 chemistry. During sequencing, libraries were automatically transferred to one lane of the flow-cell, where DNA binding occurred and clusters were generated. Clusters were then imaged as individual tiles in the flow cell prior to base-calling, filtering and quality scoring. For further details of sequencing using the Illumina MiSeq (see Ravi et al. 2018).

### Bioinformatics analysis

Reads were aligned to the revised Cambridge reference sequence (NC_012920) using the BWA-MEM algorithm (v0.7.12) and were sorted and indexed using SAMtools (v0.1.18) (Li and Durbin 2009; Li et al. 2009). The average number of reads was 32,640 per sample with 92.5% on target and an average depth of 182.1. Variant calling was performed using the Genome Analysis Toolkit (GATK; v3.2.2) with tenploidy settings that assume ten copies of the mitochondrial genome (McKenna et al. 2010). Variant calling results were input into HaploGrep2 (v2.1.0) for determination of mtDNA haplogroup (Weissensteiner et al. 2016). HaploGrep2 used PhyloTree 17 as a reference and provided a quality score for each result dependent on the reliability of the haplogroup assignment.

### Blood collection and platelet isolation

Of the 383 volunteers whose mtDNA was successfully sequenced, 30 volunteers from haplogroups *H*, *J*, *T* and *U* were recalled to donate fresh blood. Volunteers were ranked according to HaploGrep2 quality score, so that the volunteer samples with the highest haplogroup calling quality score could be selected for recall where possible. Whole blood (20 mL) was collected from each healthy volunteer in ethylenediaminetetraacetic acid (EDTA) vacutainers (BD Biosciences, USA). A tourniquet was used to assist blood collection only when absolutely required and in each case was used for < 30 s to minimise the risk of platelet activation. Within 30 min of collection, blood was layered on top of Optiprep™ density gradient medium (1.063 g/mL) using a Pasteur pipette before centrifugation (350 *g*, 15 min, no brake). Following this, the top ¾ of platelet-rich plasma (PRP) was removed and the remaining ¼ (likely to be contaminated with red blood cells) was discarded. Prostaglandin I_2_ (PGI_2_) was added to the PRP to a final concentration of 1 µg/mL to prevent platelet activation. The PRP was then centrifuged (1500 *g*, 15 min, no brake) to generate a platelet pellet. The platelet pellet was subsequently resuspended in Ca^2+^-free PBS (Life technologies, Paisley, UK) supplemented with PGI_2_ (1 µg/mL) before centrifugation (1500 *g*, 15 min, no brake).

### Platelet quantification

The absorbance of the platelet suspension, diluted tenfold, was measured (750 nm). Absorbance data were input into the following equation to quantify platelets, where k is a geometrical factor equal to 1.33 for a flat bottom 96-well microplate, *y* is the wavelength used (750 nm), R is the dilution factor of the sample (Walkowiak et al. 1997).

$$\left(\frac{6.23}{2.016-(\mathrm{k }\times \mathrm{ y }\times \left(\frac{\mathrm{reading}-\mathrm{blank}}{800}\right)} -3.09\right) \times R=platelets \times {10}^{8}/mL$$

### Platelet extracellular flux analysis

#### Preparation

Following quantification, platelets were seeded in Ca^2+^-free PBS in two CellTak (Corning™)-coated XF 96-well cell culture microplates, one for a mitochondrial stress test and one for a respiratory complex assay (1 $\times$ 10^7^ platelets/50 µL/well). Plates were then centrifuged (200 *g*, 1 min, no brake) to ensure platelet adherence.

#### Mitochondrial stress test

The PBS in one of the 96-well microplates containing platelets was replaced with unbuffered Seahorse XF base medium (175 µL/well) supplemented with glucose (25 mM), L-glutamine (2 mM), sodium pyruvate (1 mM), pre-warmed to 37 °C (pH 7.4) before incubation (1 h, 37 °C, 0% CO_2_). XF analysis was then performed as per Ball et al. 2016 using the Seahorse XFe96 Analyser. Briefly, baseline oxygen consumption rate (OCR) was measured prior to the acute injection of flutamide, 2-hydroxyflutamide or tolcapone (30, 125 or 250 µM; concentrations which have previously been used to induce mitochondrial dysfunction in the absence of whole cell death (Kamalian et al. 2015; Ball et al. 2016)). Following compound injection into the platelet medium, a mitochondrial stress test was performed utilising oligomycin (ATP synthase inhibitor; 1 µM), carbonyl cyanide 4-(trifluoromethoxy) phenylhydrazone (FCCP; uncoupler; 0.5 µM) and rotenone/antimycin A (complex I/III inhibitors, respectively; 1 µM each). This enabled the following parameters to be measured: basal, maximum and ATP-linked respiration, spare respiratory capacity and proton leak (see Ball et al. 2016 for derivation of parameters).

#### Respiratory complex assays

These assays were performed as described previously (Ball et al. 2016). Briefly, PBS was replaced with mitochondrial assay solution buffer (MAS: MgCl_2_; 5 mM, mannitol; 220 mM, sucrose; 70 mM, KH_2_PO_4_; 10 mM, HEPES; 2 mM, EGTA; 1 mM, bovine serum albumin (BSA); 0.4% w/v) containing constituents to permeabilise cells and stimulate oxygen consumption via complex I (ADP; 4.6 mM, malic acid; 30 mM, glutamic acid; 22 mM, BSA; 30 µM, PMP; 1 nM), complex II (ADP; 4.6 mM, succinic acid; 20 mM, rotenone; 1 µM, BSA; 30 µM, PMP; 1 nM), complex III (ADP; 4.6 mM, duroquinol; 500 µM, rotenone; 1 µM, malonic acid; 40 µM, BSA; 0.2% w/v, PMP; 1 nM) or complex IV (ADP; 4.6 mM, ascorbic acid; 20 mM, N,N,N′,N′-tetramethyl-p-phenylenediamine (TMPD); 0.5 mM, antimycin A; 2 mM, BSA; 30 mM, PMP; 1 nM). Following a basal measurement period (2-hydroxyflutamide was injected (15, 30, 125 or 250 µM) followed by a mitochondrial stress test. For assessment of complex IV-driven respiration, potassium azide (complex IV inhibitor), rather than rotenone/antimycin A (complex I and III inhibitors, respectively), was injected to prevent mitochondrial respiration.

### Statistical analysis

Results from the 30 healthy volunteers were pooled according to haplogroup; *H* (*n* = 8), *J* (*n* = 6), *T* (*n* = 7) and *U* (*n* = 9). Results from mitochondrial stress tests and complex assays were assessed for statistically significant differences between haplogroups. The following parameters were selected for comparison: basal, maximum and ATP-linked respiration, spare respiratory capacity, proton leak and complex I–IV-driven respiration.

Pearson’s correlation coefficient was calculated to establish if parameters of basal mitochondrial function: spare respiratory capacity, basal respiration; could be used to predict outputs of dysfunction: EC_50_ ATP-linked respiration (flutamide, 2-hydroxyflutamide, tolcapone) and if basal complex I/II-driven respiration could be used to predict 2-hydroxyflutamide EC_50_ complex I/II-driven respiration. Results from platelets of all haplogroups were pooled to provide a sufficient *n* number for the correlation analysis. EC_50_ values were determined by nonlinear regression analysis using GraphPad Prism 7.0 following mean centring using SPSS v24. Normality was assessed using a Shapiro–Wilk test. Statistical significance was determined by a one-way ANOVA for parametric data followed by a Dunnett’s post-hoc test using StatsDirect 2.7.9. Differences were determined to be significant at *p* < 0.05.

## Results

### Phylogenetic distribution of samples

**Fig. 1:**
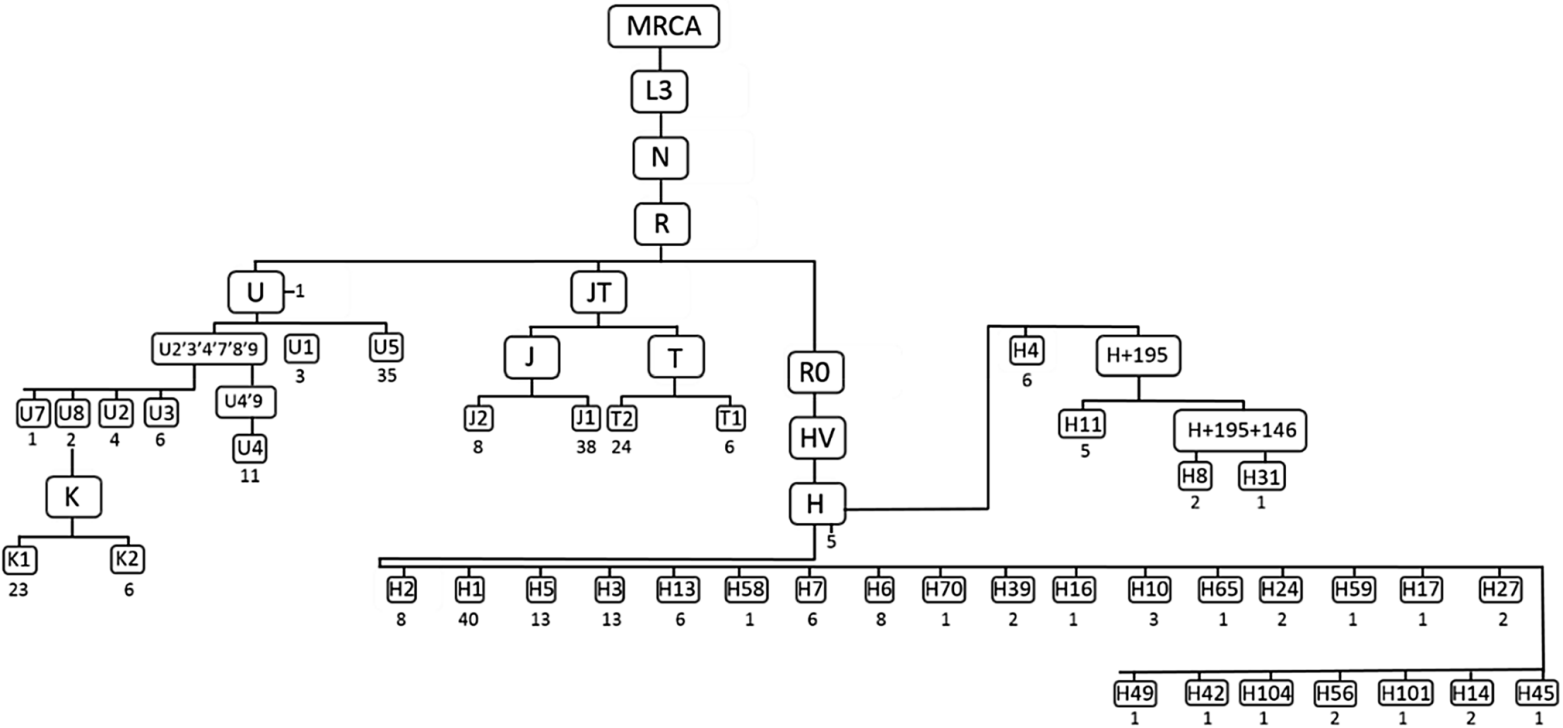
Phylogenetic tree of the five most common haplogroups in the study cohort. Genotyping results from 383 healthy volunteers were classified into mitochondrial sub-haplogroups; the five most common haplogroups are displayed here. The number of samples is displayed under each group. *MRCA* most recent common ancestor

383 healthy volunteer samples were sequenced successfully. As well as determining haplogroup classification and quality information, HaploGrep2 provided the resultant phylogenetic tree of all samples (Supplementary Fig. 3). A simplified version of this phylogenetic tree is displayed in Fig. 1. Haplogroup assignment and SNPs of the 30 volunteers who donated blood for subsequent platelet function studies are detailed in Supplementary Fig. 4.

### Basal mitochondrial respiratory function

**Fig. 2:**
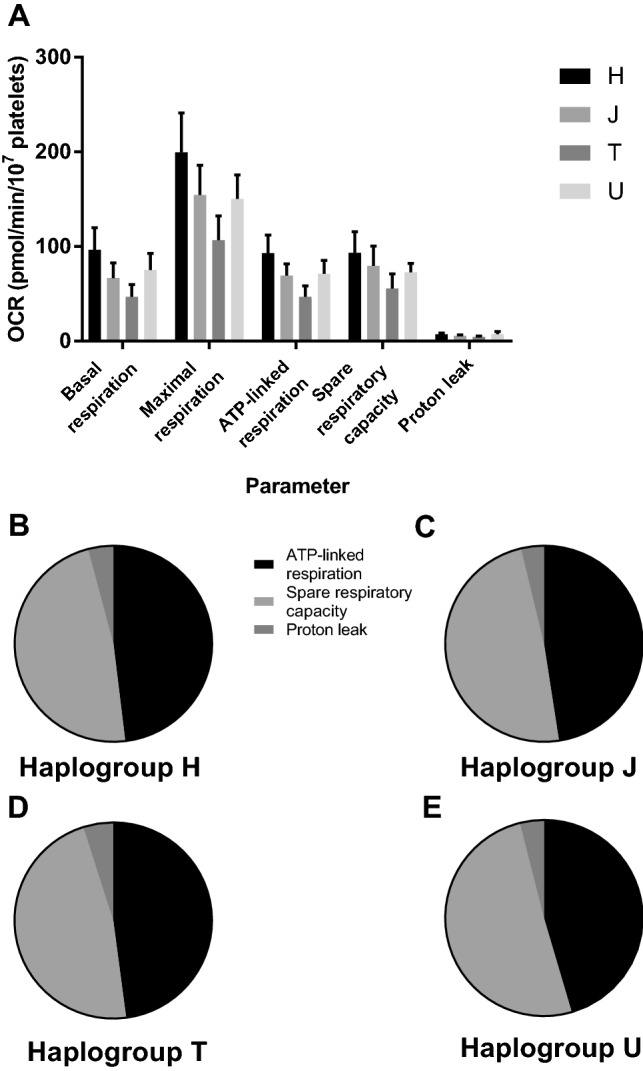
Basal mitochondrial function. Extracellular flux analysis was used to perform a mitochondrial stress test using platelets from donors of haplogroups *H*, *J*, *T* and *U*. **a** basal, maximal and ATP-linked respiration, spare respiratory capacity and proton leak of platelets from each haplogroup. **b–e**: ATP-linked respiration, spare respiratory capacity and proton leak as a proportion of maximal respiration. There were no statistically significant differences between haplogroups. Data are presented as mean + SEM of n ≥ 6 independent experiments. *OCR* oxygen consumption rate

Assessment of basal mitochondrial respiratory function using a mitochondrial stress test identified increased respiration in haplogroup *H* and *U* platelets compared with haplogroups *J* and *T*, though this did not reach statistical significance (Fig. 2a). When each parameter was considered as a proportion of maximal respiration, there was no significant difference in ATP-linked respiration, spare respiratory capacity and proton leak between haplogroups (Fig. 2b–e).

**Fig. 3:**
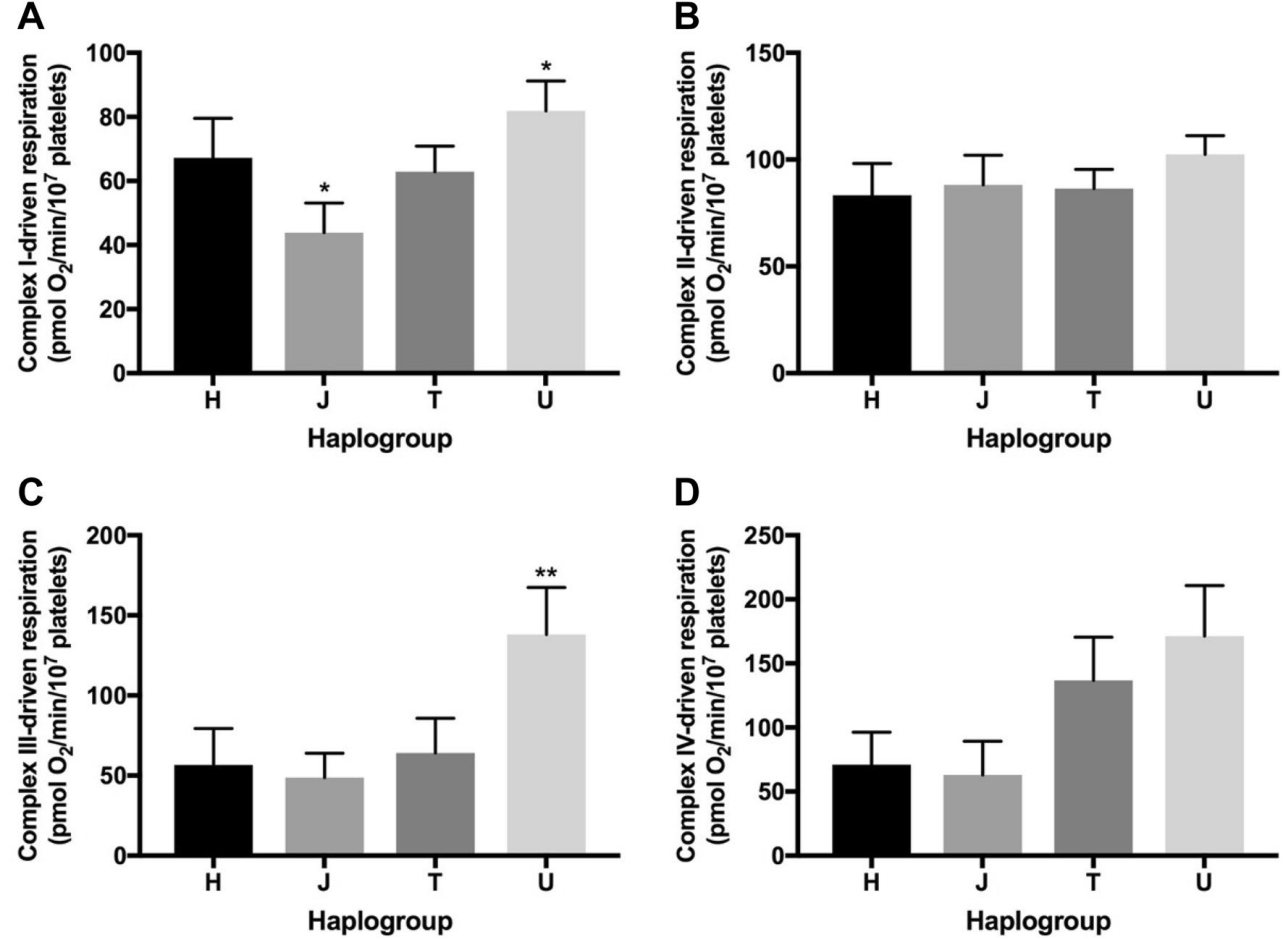
Basal complex-driven respiration. Extracellular flux analysis of platelets from donors of haplogroups *H*, *J*, *T* and *U* was performed using uncoupled, permeabilised platelets, with respiration driven via the addition of substrates specific to complexes I (**a**), II (**b**), III (**c**) or IV (**d**). Complex-driven respiration was defined by the maximal respiration when treated with substrates specific to the complex of interest. Statistical significance compared with other haplogroups, e.g., H vs. non-H: *p < 0.05, **p < 0.01, ***p < 0.001. Data are presented as mean ± SEM of *n* ≥ 6 independent experiments

In permeabilised platelets, complex I-driven respiration was significantly lower in haplogroup *J* platelets (Fig. 3a), whereas haplogroup *U* platelets exhibited significantly increased complex I- and III-driven respiration compared with the remaining haplogroups (Fig. 3a, c). Haplogroup *U* platelets also had the greatest complex IV-driven respiration (Fig. 3d). Complex II-driven respiration was similar across platelets of all haplogroups (Fig. 3b).

### Flutamide-, 2-hydroxyflutamide- and tolcapone-treated mitochondrial respiration

**Fig. 4:**
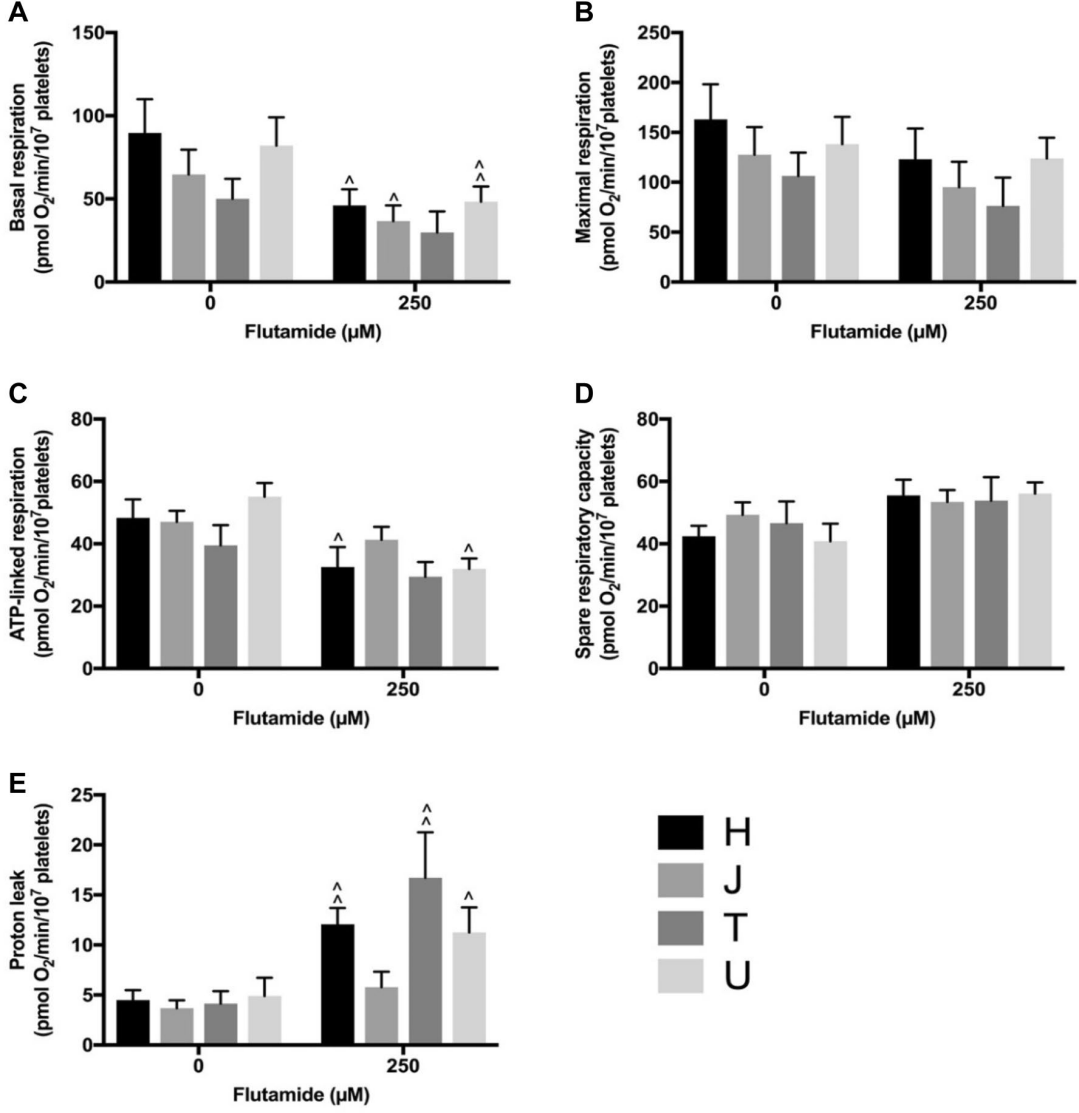
Mitochondrial respiratory function of flutamide-treated platelets. Extracellular flux analysis of platelets from donors of haplogroups *H*, *J*, *T* and *U* was performed following acute treatment with flutamide. Changes in basal respiration (**a**), maximal respiration (**b**), ATP-linked respiration (**c**), spare respiratory capacity (**d**) and proton leak (**e**) were measured. Statistical significance vs. vehicle control: ^*p* < 0.05, ^^*p* < 0.01, ^^^*p* < 0.001. There were no statistically significant differences between haplogroups. For clarity, only data from 250 μM treatment are shown (see Supplementary Fig. 6 for full concentration range) Data are presented as mean + SEM of *n* ≥ 6 independent experiments

**Fig. 5:**
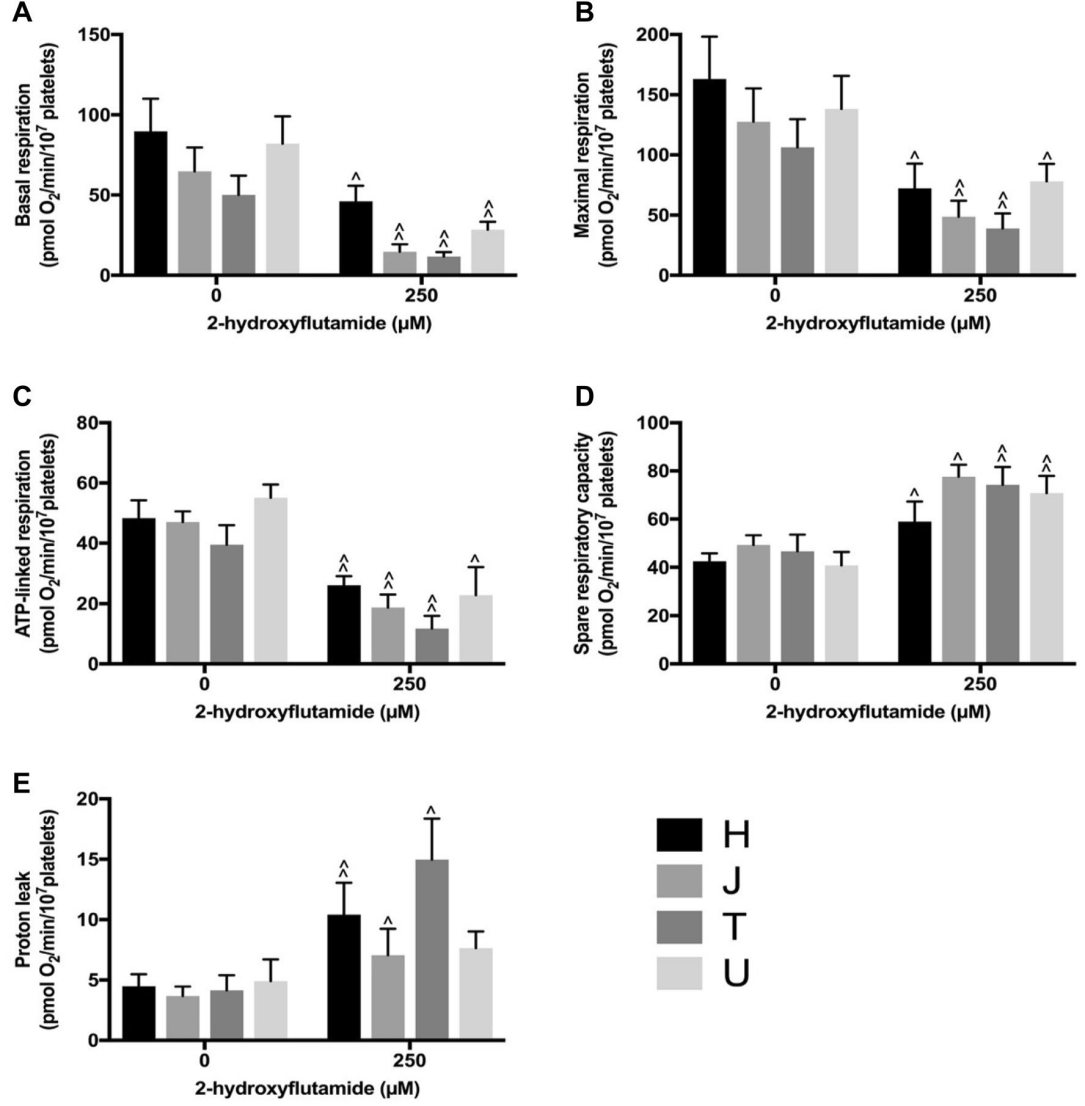
Mitochondrial respiratory function of 2-hydroxyflutamide-treated platelets. Extracellular flux analysis of platelets from donors of haplogroups *H*, *J*, *T* and *U* was performed following acute treatment with 2-hydroxyflutamide. Changes in basal respiration (**a**), maximal respiration (**b**), ATP-linked respiration (**c**), spare respiratory capacity (**d**) and proton leak (**e**) were measured. Statistical significance vs. vehicle control: ^*p* < 0.05, ^^*p* < 0.01, ^^^*p* < 0.001. There were no statistically significant differences between haplogroups. For clarity, only data from 250 μM treatment are shown (see Supplementary Fig. 7 for full concentration range). Data are presented as mean + SEM of *n* ≥ 6 independent experiments

**Fig. 6:**
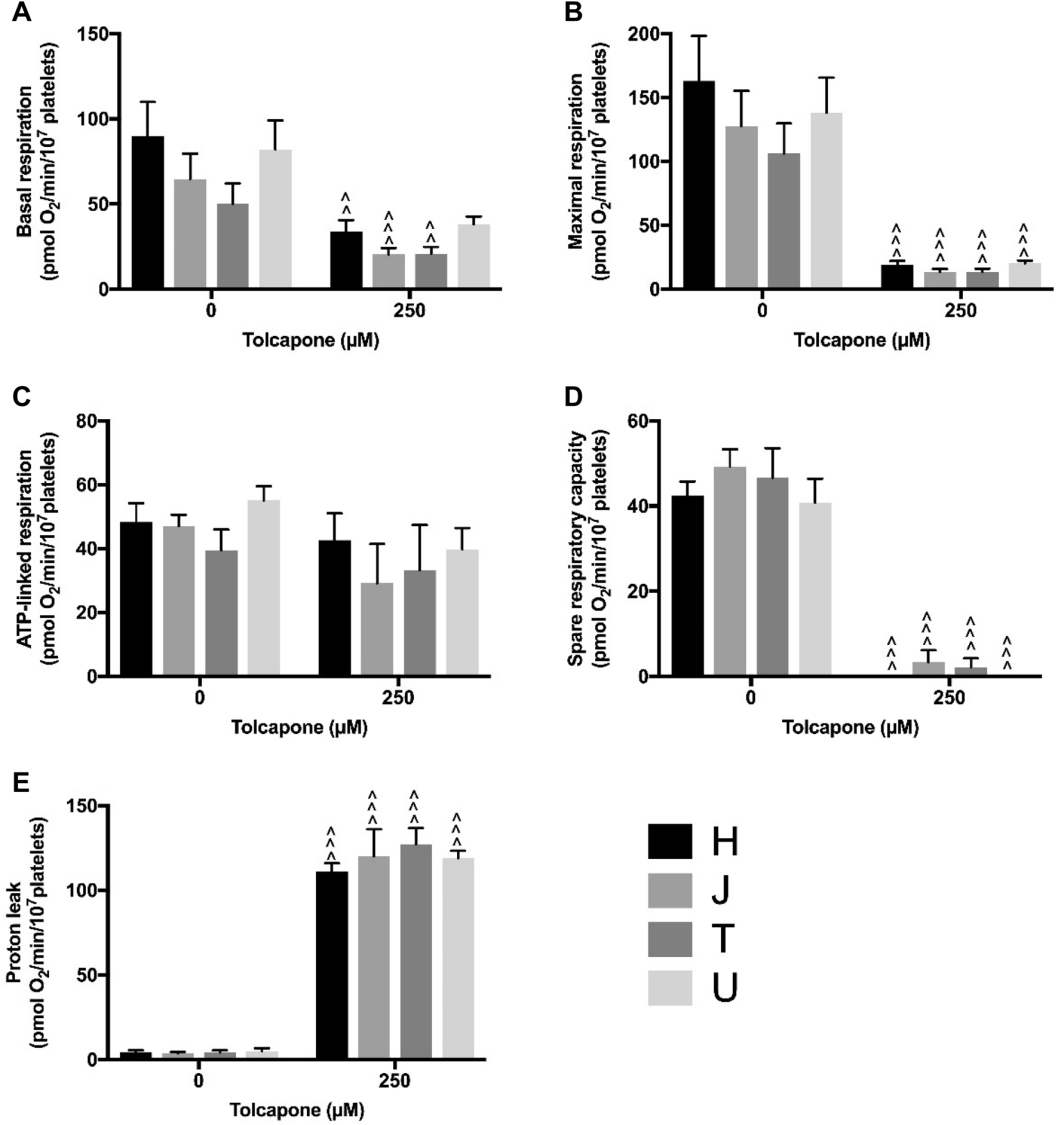
Mitochondrial respiratory function of tolcapone-treated platelets. Extracellular flux analysis of platelets from donors of haplogroups *H*, *J*, *T* and *U* was performed following acute treatment with tolcapone. Changes in basal respiration (**a**), maximal respiration (**b**), ATP-linked respiration (**c**), spare respiratory capacity (**d**) and proton leak (**e**) were measured. It should be noted that there is a substantial difference in the scale of the y axis between Fig. 6e and Figs. 4e and 5e. Statistical significance vs. vehicle control: ^*p* < 0.05, ^^*p* < 0.01, ^^^*p* < 0.001. There were no statistically significant differences between haplogroups. For clarity only data from 250 μM treatment are shown (see Supplementary Fig. 8 for full concentration range). Data are presented as mean + SEM of *n* ≥ 6 independent experiments

Multiple parameters of mitochondrial function were measured following acute treatment of platelets with 250 μM flutamide (Fig. 4), 2-hydroxyflutamide (Fig. 5) or tolcapone (Fig. 6). No significant differences between haplogroups were identified and generally, inter-haplogroup variation upon treatment with 250 μM of each compound was in-line with inter-haplogroup variation with control treatment. Notably, haplogroup *T* platelets demonstrated the greatest increase in proton leak when treated with 250 μM flutamide or 2-hydroxyflutamide. For the results of mitochondrial stress tests using 10, 30 and 125 μM compound concentrations see Supplementary Figs. 6, 7, 8.

### 2-hydroxyflutamide-induced respiratory complex I and II dysfunction

**Fig. 7:**
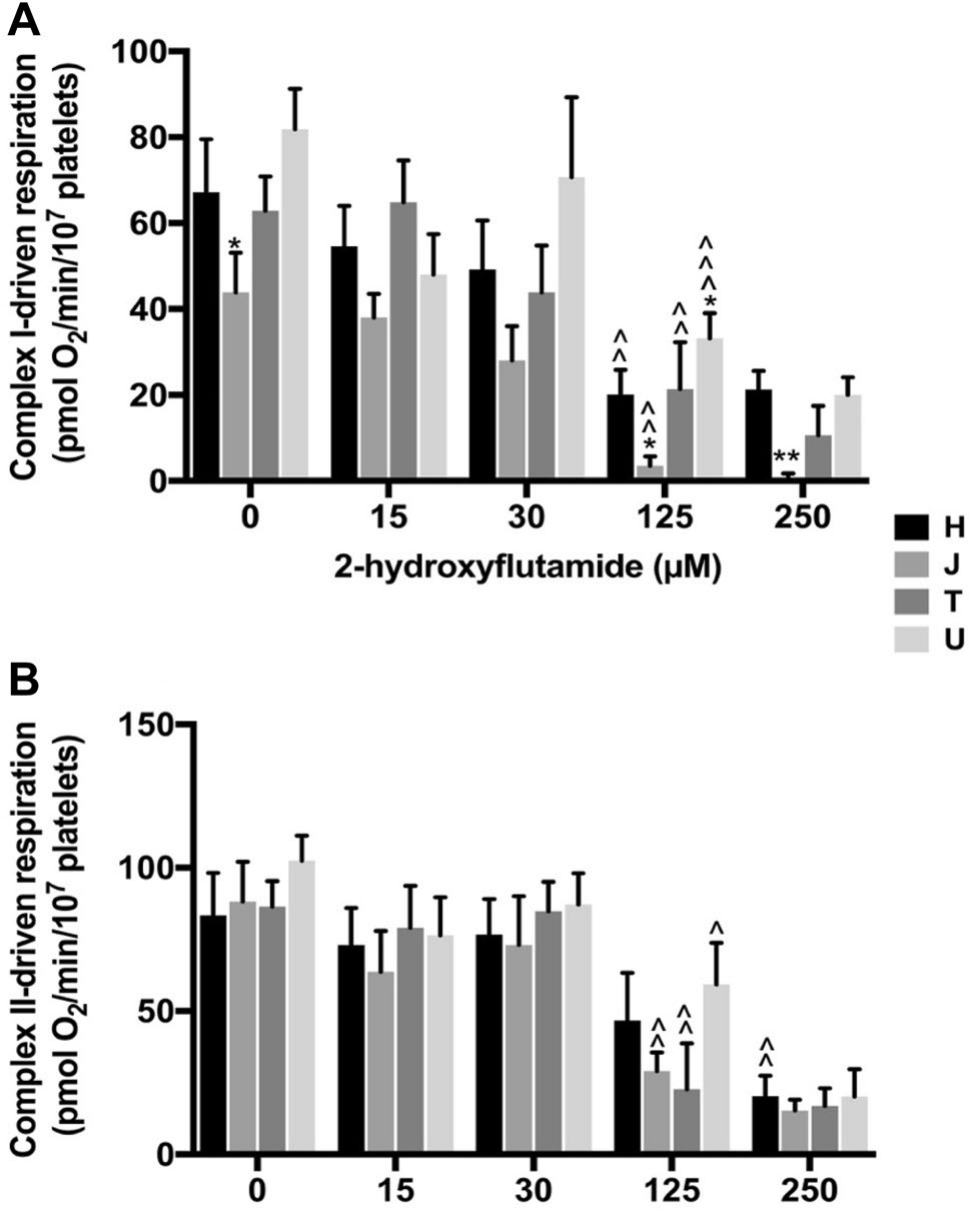
Complex I and II-driven respiration in 2-hydroxyflutamide-treated platelets. Extracellular flux analysis of permeabilised platelets from donors of haplogroups *H*, *J*, *T* and *U* was performed in solution containing substrates specific to complexes I (**a**), II (**b**) following acute treatment with 2-hydroxyflutamide. Statistical significance vs. vehicle control: ^*p* < 0.05, ^^*p* < 0.01, ^^^*p* < 0.001. For clarity only the first point of significance are shown. Statistical significance compared with other haplogroups, e.g., H vs. non-H: *p < 0.05, **p < 0.01, ***p < 0.001. Data are presented as mean + SEM of *n* ≥ 6 independent experiments

Haplogroup *J* platelets had significantly lower complex I-driven respiration both when untreated and when treated with 250 μM 2-hydroxyflutamide. This was also reflected in the EC_50_ for complex I-driven respiration in haplogroup *J* platelets, which was significantly less than the remaining haplogroups (Table 1). Haplogroup *U* platelets had consistently  greater complex I-driven respiration, which reached significance at 125 μM (Fig. 7a).

**Table 1:** EC_50_ complex I and II-driven respiration of 2-hydroxyflutamide in platelets from donors of haplogroups *H*, *J*, *T* and *U*

Complex	EC_50_ complex-driven respiration (µM)			
	*H*	*J*	*T*	*U*
I	118 ± 16.2	49.3 ± 5.55 (0.0390)	69.0 ± 9.72	70.8 ± 9.47
II	144 ± 12.1	63.1 ± 3.92 (0.0200)	79.0 ± 8.96	110 ± 8.80

EC_50_ complex-driven respiration refers to the concentration of 2-hydroxyflutamide required to reduce complex I/II-driven respiration by 50%. Data are presented as mean ± SEM (*p* value) of *n* ≥ 6 independent experiments. *P* value indicates statistical significance compared with other haplogroups and is only shown if < 0.05.

Complex II-driven respiration was similar in all haplogroups, except upon treatment with 125 μM 2-hydroxyflutamide, where haplogroup *U* had a higher activity. When treated with 250 μM 2-hydroxyflutamide, all haplogroups demonstrated a similar reduction in activity (Fig. 7b).

### Correlation between basal and treated mitochondrial respiratory function

The ability of basal values of mitochondrial function to predict responses to compound treatment was determined by derivation of the correlation coefficient between basal and treated outputs of mitochondrial function (Table 2). The spare respiratory capacity of untreated platelets provided the strongest positive correlation (*R* value nearest to +1) with the EC_50_ ATP-linked respiration of flutamide and 2-hydroxyflutamide, but not tolcapone. Basal respiration and complex I/II-driven respiration, however, failed to strongly correlate (*R* value near 0) with the EC_50_ of any of the test compounds.

**Table 2:** Correlation of basal mitochondrial function with flutamide-, 2-hydroxyflutamide- and tolcapone-treated mitochondrial function

Variable 1	Variable 2	*R*	*P* value
Complex I-driven respiration	2-Hydroxyflutamide EC_50_ complex I-driven respiration	0.23	0.3356
Complex II-driven respiration	2-Hydroxyflutamide EC_50_ complex II-driven respiration	0.33	0.4010
Spare respiratory capacity	Flutamide EC_50_ ATP-linked respiration	0.72	< 0.0001
Spare respiratory capacity	2-Hydroxyflutamide EC_50_ ATP-linked respiration	0.63	0.0002
Spare respiratory capacity	Tolcapone EC_50_ ATP-linked respiration	0.35	0.0602
Basal respiration	Flutamide EC_50_ ATP-linked respiration	0.17	0.3622
Basal respiration	2-Hydroxyflutamide EC_50_ ATP-linked respiration	0.28	0.1278
Basal respiration	Tolcapone EC_50_ ATP-linked respiration	0.18	0.3446

*Variable 1* functions of platelets in a basal state, *Variable 2* functions of platelets in a treated state, *R* Pearson’s correlation coefficient, *P* *value* statistical significance of correlation

## Discussion

The abundance of mtDNA and absence of nuclear DNA in platelets makes these cell fragments an attractive model to study the effect of mtDNA variation upon mitochondrial function. In this study, the mitochondrial haplogroup of 383 healthy volunteers was determined using whole mitochondrial genome sequencing. This information was used to select individuals from the most common mitochondrial haplogroups in England; *H*, *J*, *T* and *U*; whom were recalled to donate platelets. This enabled mitochondrial function differences between haplogroups to be assessed in platelets at basal state and in platelets treated with therapeutic compounds known to induce mitochondrial dysfunction; flutamide, 2-hydroxyflutamide (primary metabolite of flutamide) and tolcapone.

Total mitochondrial respiration did not vary significantly between haplogroups, though haplogroup *J* and *T* platelets had lower respiration compared with haplogroups *H* and *U* across multiple treatment doses. Treatment of permeabilised platelets provided with respiratory complex-specific substrates enabled inter-haplogroup differences to be assessed at the sub-mitochondrial level. Here, haplogroup *J* platelets had significantly lower complex I-driven respiration both when untreated and when treated with 250 μM 2-hydroxyflutamide. This was also reflected in the EC_50_ complex I-driven respiration of 2-hydroxyflutamide in haplogroup *J* platelets, which was significantly less than the remaining haplogroups. The reduced complex I-driven respiration of haplogroup *J* platelets could be associated with the non-synonymous SNPs characteristic of this haplogroup including A10398G (rs2853826) and G13708A (rs28359178) in regions encoding complex I subunits, MT-ND3 and MT-ND5, respectively (Czarnecka et al. 2010; Pignataro et al. 2017). Work utilising transmitochondrial cybrids has noted that these mutations result in a significant difference in ATP-production driven specifically via complex I/II, between haplogroups *J* and *U*, which mirrors the current findings (Ghelli et al. 2009). Three of the haplogroup *J* donor samples used in this study (9, 16 and 30; see Supplementary Fig. 4) also contained the T3394C SNP within the region encoding MT-ND1 (complex I), characteristic of the J1c1 sub-haplogroup (Liang et al. 2009).

The fact that significant differences in total mitochondrial respiration between haplogroups were not observed may be a product of the limited size of this cohort and the heterogeneity encompassed within haplogroups. However, it should be noted that work by Strobbe et al. using transmitochondrial cybrids of haplogroups *H*, *J*, *T*, *U* and *K* has also reported little variance in basal bioenergetic function but significant differences at the respiratory complex level when using rotenone as a complex-I specific inhibitor. Specifically, haplogroup *J* was found to be more sensitive, in agreement with the overall findings of the current study (Strobbe et al. 2018).

In contrast to complex I, complex II is entirely encoded in the nuclear genome; therefore, a difference in complex II activity between haplogroups would not be anticipated; indeed this was the case in this work, with no significant difference in platelet complex II-driven respiration between haplogroups (Chinnery and Hudson 2013). Overall, the concurrence in the findings presented in this manuscript with other studies in the field provides additional confidence in the utility of platelets as an ex vivo model for studying the effect of mitochondrial genotype on susceptibility to drug-induced mitochondrial dysfunction.

This study also pooled the results from all haplogroups to investigate if the extent of drug-induced mitochondrial dysfunction could be predicted from the assessment of mitochondrial function at basal state (untreated). Spare respiratory capacity was not significantly different between haplogroups in untreated platelets, but was significantly positively correlated with the EC_50_ of ATP-linked respiration in flutamide and 2-hydroxyflutamide-treated platelets, i.e., platelets with increased spare respiratory capacity were observed to have greater resistance to the decrease in ATP-linked respiration induced by flutamide and 2-hydroxyflutamide. This observation could be due to spare respiratory capacity which is able to mitigate against 2-hydroxyflutamide-induced ETC disruption which results in the higher EC_50_. This constitutes an important finding as it demonstrates the potential utility of platelets for the prediction of drug-induced mitochondrial dysfunction based on basal state measurements. To reach the EC_50_ and observe these correlations, the concentrations used greatly exceeded the C_max_ of the compounds (flutamide; 72.2 nM, 2-hydroxyflutamide; 4.4 μM, tolcapone; 16.5 μM); however, these concentrations have previously been used to induce mitochondrial dysfunction in the absence of whole cell death (Kamalian et al. 2015; Ball et al. 2016).

Previous research has questioned the suitability of platelets for such work due to a reported lack of spare respiratory capacity, indicating that cells are functioning at their near maximal energetic capacity under basal conditions (Chacko et al. 2013). However, in the current study, platelets were demonstrated to be a practical, primary cell model, which is amenable to the study of drug-induced mitochondrial dysfunction. Specifically, the results reported here, where platelets were typically analysed 2.5 h after blood samples were taken, demonstrate that freshly-isolated platelets retain mitochondrial function post their isolation from blood samples and possess significant spare respiratory capacity. This study has also demonstrated the reproducibility of bioenergetic assessments in platelet models by measuring mitochondrial respiration in platelets from two volunteers (of unknown haplogroup) across a period of 5 days (blood collected and platelets analysed on days 0, 2 and 4). Although maximal respiration of platelets varied between each day, there were negligible changes in parameters of mitochondrial respiratory function as a proportion of maximal respiration (Supplementary Fig. 5).

To conclude, this research has indicated that platelets are a potentially valuable ex vivo model of inter-individual variation in mtDNA and susceptibility to drug-induced mitochondrial dysfunction induced by compounds associated with adverse drug reactions. Specifically, platelets from donors of haplogroup *J* have been found to have increased susceptibility to the acute inhibition of complex I-driven respiration by 2-hydroxyflutamide. However, in many of the tests performed, variation did not reach statistical significance, a potential artefact of the heterogeneity of mitochondrial haplogroups (Supplementary Fig. 4). Nonetheless, at a time when individual susceptibility to adverse drug reactions is not fully understood, this study provides evidence that inter-individual variation in mitochondrial genotype can be a factor in determining sensitivity to mitochondrial toxicants and will serve as a foundation for future investigations.

## Supplementary Information

Below is the link to the electronic supplementary material.

Supplementary Figure 1: Cycling conditions for multiplex amplicon tagging (TIF 65 kb)

Supplementary Figure 2: Conditions for incorporation of Illumina barcodes (TIFF 15 kb)

Supplementary Figure 3: Phylogenetic tree of 383 healthy volunteers (PNG 7607 kb)

Supplementary Figure 4: Mitochondrial DNA variation of 30 healthy volunteers who donated platelets (TIF 1010 kb)

Supplementary Figure 5: Day-to-day variation of platelet mitochondrial function. Two healthy volunteers (of unknown mitochondrial genotype) donated platelets for extracellular flux analysis on days 0, 2 and 4. Values for parameters, ATP-linked respiration, spare respiratory capacity and proton leak are presented as a percentage of the maximal respiration in the same experiment. Coupling efficiency (the proportion of the oxygen consumed to drive ATP synthesis ) was calculated by ATP-linked respiration/basal respiration × 100. Data are presented as mean + SEM of n = 1 experiments (30 technical replicates) (TIF 464 kb)

Supplementary Figure 6: Mitochondrial function of flutamide-treated platelets. Extracellular flux analysis of platelets from donors of haplogroups H, J, T and U was performed following acute treatment with flutamide. Changes in basal respiration (A), maximal respiration (B), ATP-linked respiration (C), spare respiratory capacity (D) and proton leak (E). Statistical significance compared to vehicle control: ^ p < 0.05, ^^ p < 0.01, ^^^ p < 0.001. For clarity only the first point of significance is shown. Data are presented as mean + SEM of n ≥ 6 independent (TIF 308 kb)

Supplementary Figure 7 Mitochondrial function of 2-hydroxyflutamide-treated platelets. Extracellular flux analysis of platelets from donors of haplogroups H, J, T and U was performed following acute treatment with 2-hydroxyflutamide. A, B: changes in maximal and basal respiration, C-E: changes in ATP-linked respiration, spare respiratory capacity and proton leak respectively. Statistical significance compared to vehicle control: ^ p < 0.05, ^^ p < 0.01, ^^^ p < 0.001. For clarity only the first point of significance is shown. Data are presented as mean + SEM of n ≥ 6 independent experiments (TIFF 307 kb)

Supplementary Figure 8: Mitochondrial function of tolcapone-treated platelets. Extracellular flux analysis of platelets from donors of haplogroups H, J, T and U was performed following acute treatment with tolcapone. A, B: changes in maximal and basal respiration, C-E: changes in ATP-linked respiration, spare respiratory capacity and proton leak respectively. Statistical significance compared to vehicle control: ^ p < 0.05, ^^ p < 0.01, ^^^ p < 0.001. For clarity only the first point of significance is shown. Statistical significance of haplogroups J, T compared to other haplogroups; + p < 0.05. Data are presented as mean + SEM of n ≥ 6 independent experiments (TIF 292 kb)
