# Supplementary figures and images for: Assessment of the impact of mitochondrial genotype upon drug-induced mitochondrial dysfunction in platelets derived from healthy volunteers
Source: Arch Toxicol. 2021 Feb 13;95(4):1335–47. doi: 10.1007/s00204-021-02988-3 (PMC8032628; doi:10.1007/s00204-021-02988-3)

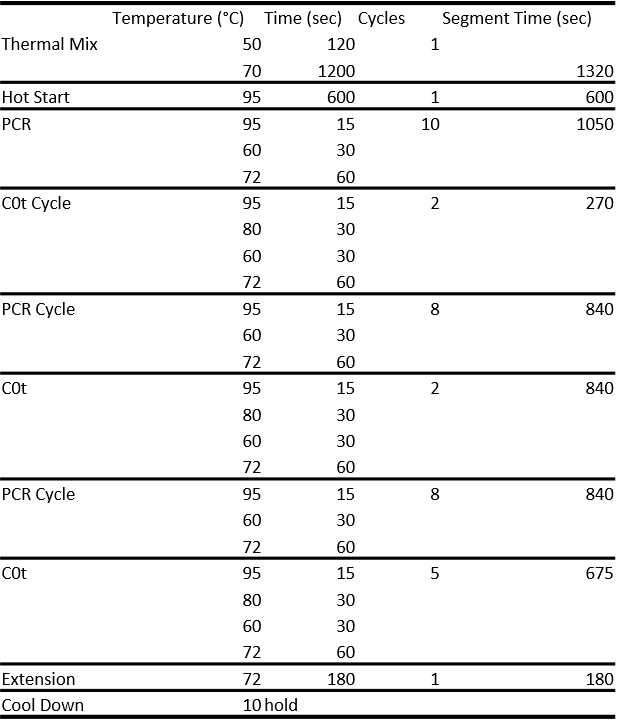

Supplement: Supplementary file 1 — Supplementary Figure 1: Cycling conditions for multiplex amplicon tagging (TIF 65 kb) [file 204_2021_2988_MOESM1_ESM.tif]

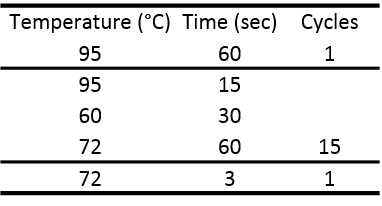

Supplement: Supplementary file 2 — Supplementary Figure 2: Conditions for incorporation of Illumina barcodes (TIFF 15 kb) [file 204_2021_2988_MOESM2_ESM.tif]

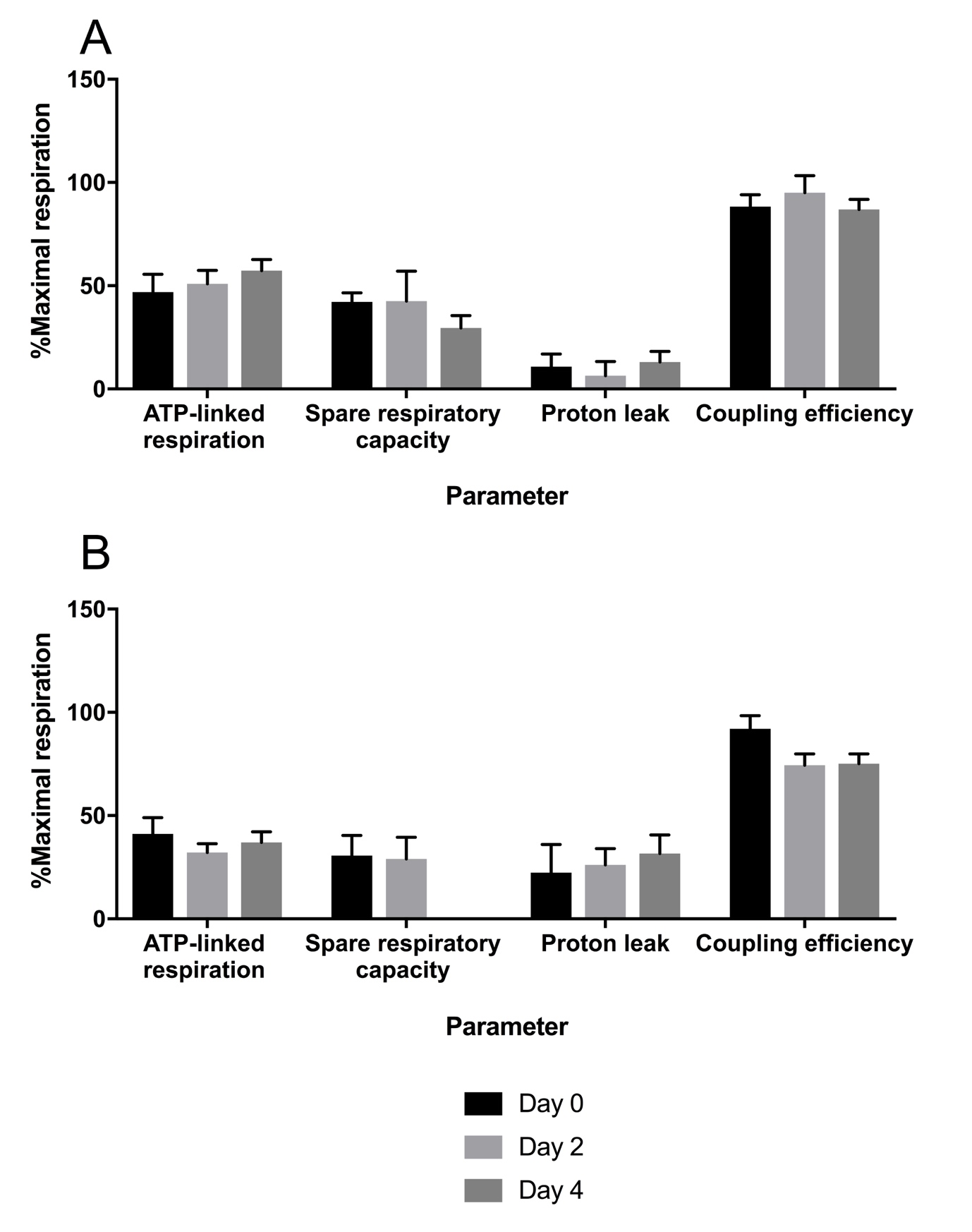

Supplement: Supplementary file 5 — Supplementary Figure 5: Day-to-day variation of platelet mitochondrial function. Two healthy volunteers (of unknown mitochondrial genotype) donated platelets for extracellular flux analysis on days 0, 2 and 4. Values for parameters, ATP-linked respiration, spare respiratory capacity and proton leak are presented as a percentage of the maximal respiration in the same experiment. Coupling efficiency (the proportion of the oxygen consumed to drive ATP synthesis ) was calculated by ATP-linked respiration/basal respiration × 100. Data are presented as mean + SEM of n = 1 experiments (30 technical replicates) (TIF 464 kb) [file 204_2021_2988_MOESM5_ESM.tif]

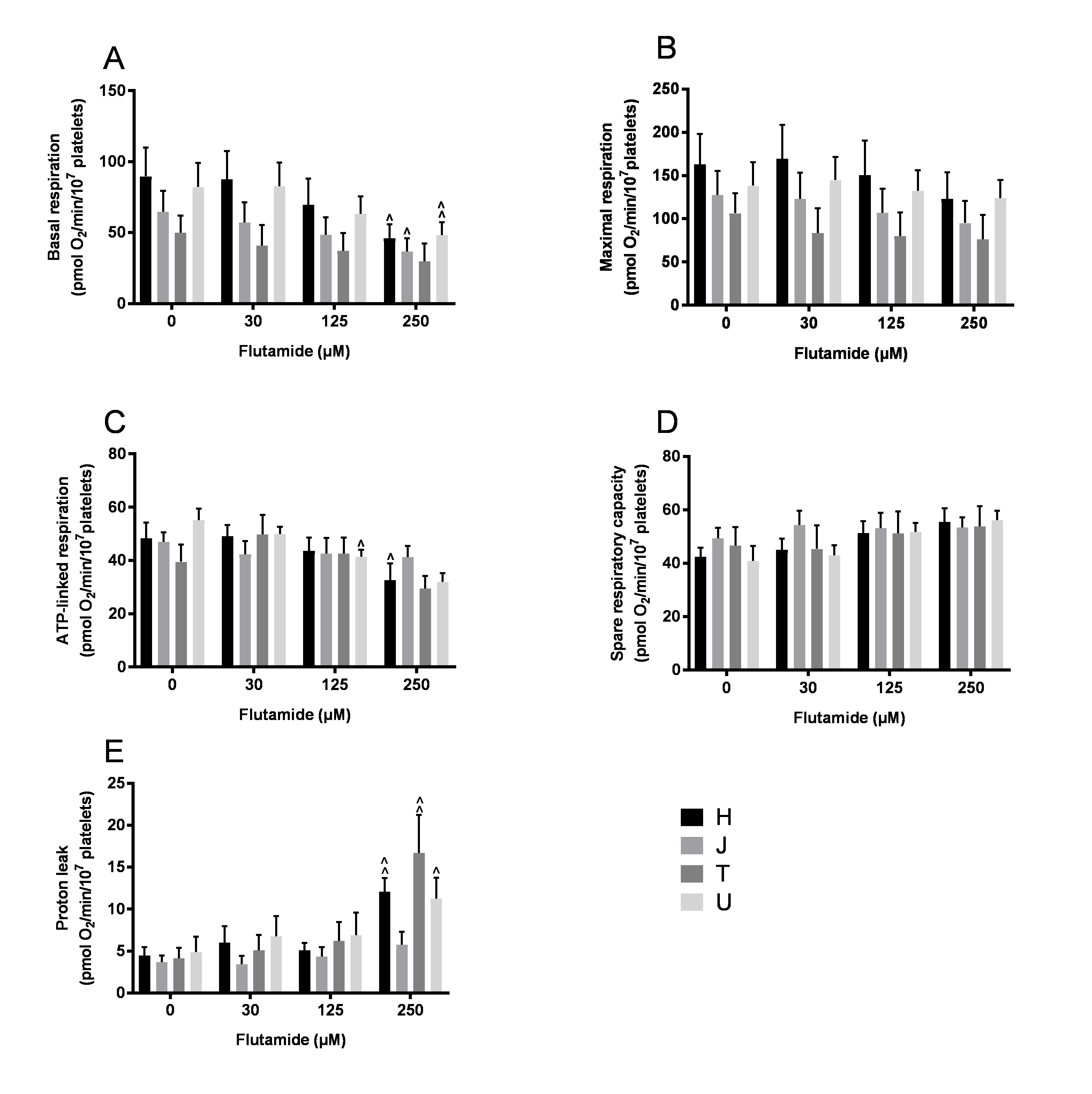

Supplement: Supplementary file 6 — Supplementary Figure 6: Mitochondrial function of flutamide-treated platelets. Extracellular flux analysis of platelets from donors of haplogroups H, J, T and U was performed following acute treatment with flutamide. Changes in basal respiration (A), maximal respiration (B), ATP-linked respiration (C), spare respiratory capacity (D) and proton leak (E). Statistical significance compared to vehicle control: ^ p < 0.05, ^^ p < 0.01, ^^^ p < 0.001. For clarity only the first point of significance is shown. Data are presented as mean + SEM of n ≥ 6 independent (TIF 308 kb) [file 204_2021_2988_MOESM6_ESM.tif]

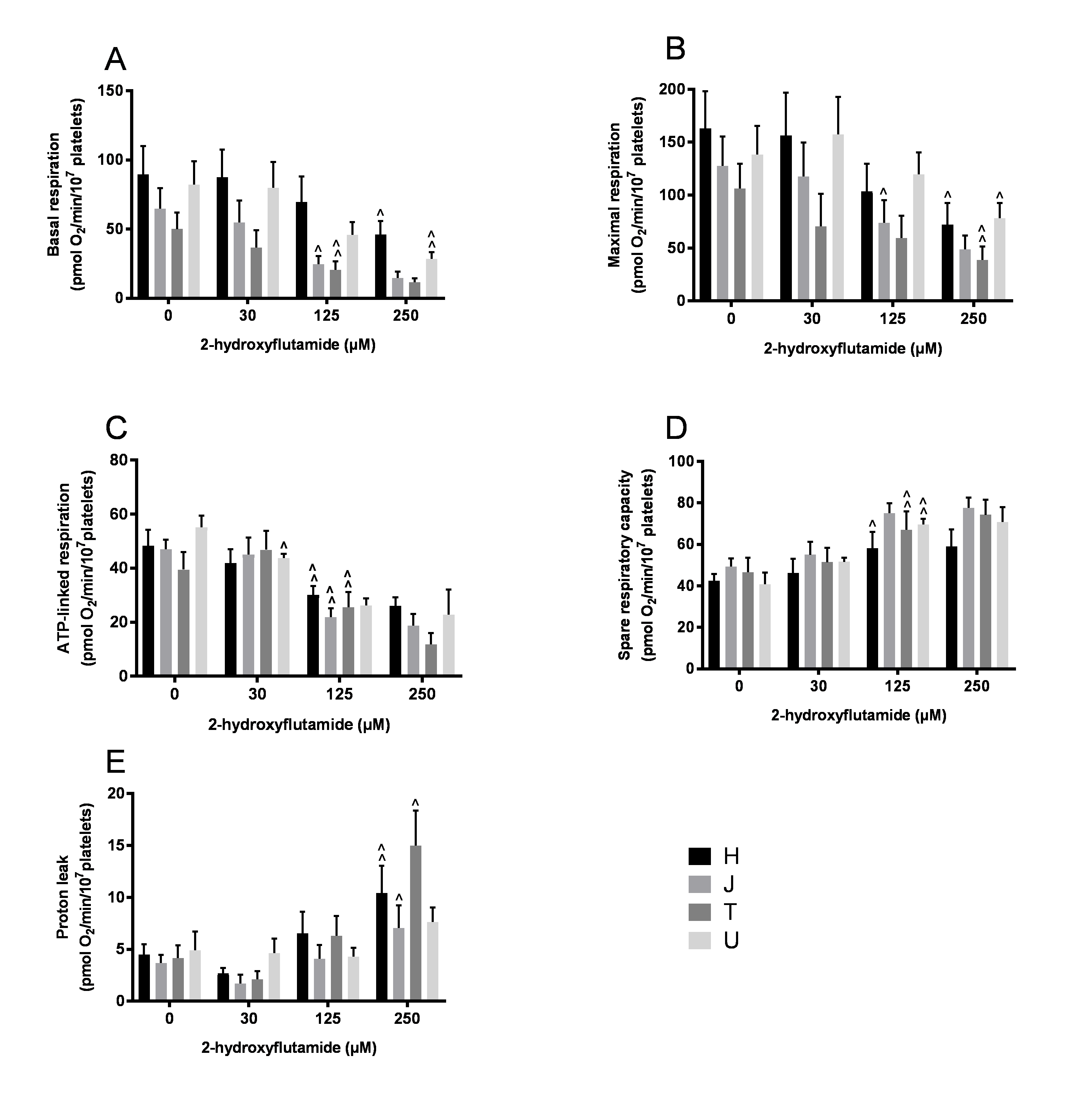

Supplement: Supplementary file 7 — Supplementary Figure 7 Mitochondrial function of 2-hydroxyflutamide-treated platelets. Extracellular flux analysis of platelets from donors of haplogroups H, J, T and U was performed following acute treatment with 2-hydroxyflutamide. A, B: changes in maximal and basal respiration, C-E: changes in ATP-linked respiration, spare respiratory capacity and proton leak respectively. Statistical significance compared to vehicle control: ^ p < 0.05, ^^ p < 0.01, ^^^ p < 0.001. For clarity only the first point of significance is shown. Data are presented as mean + SEM of n ≥ 6 independent experiments (TIFF 307 kb) [file 204_2021_2988_MOESM7_ESM.tif]

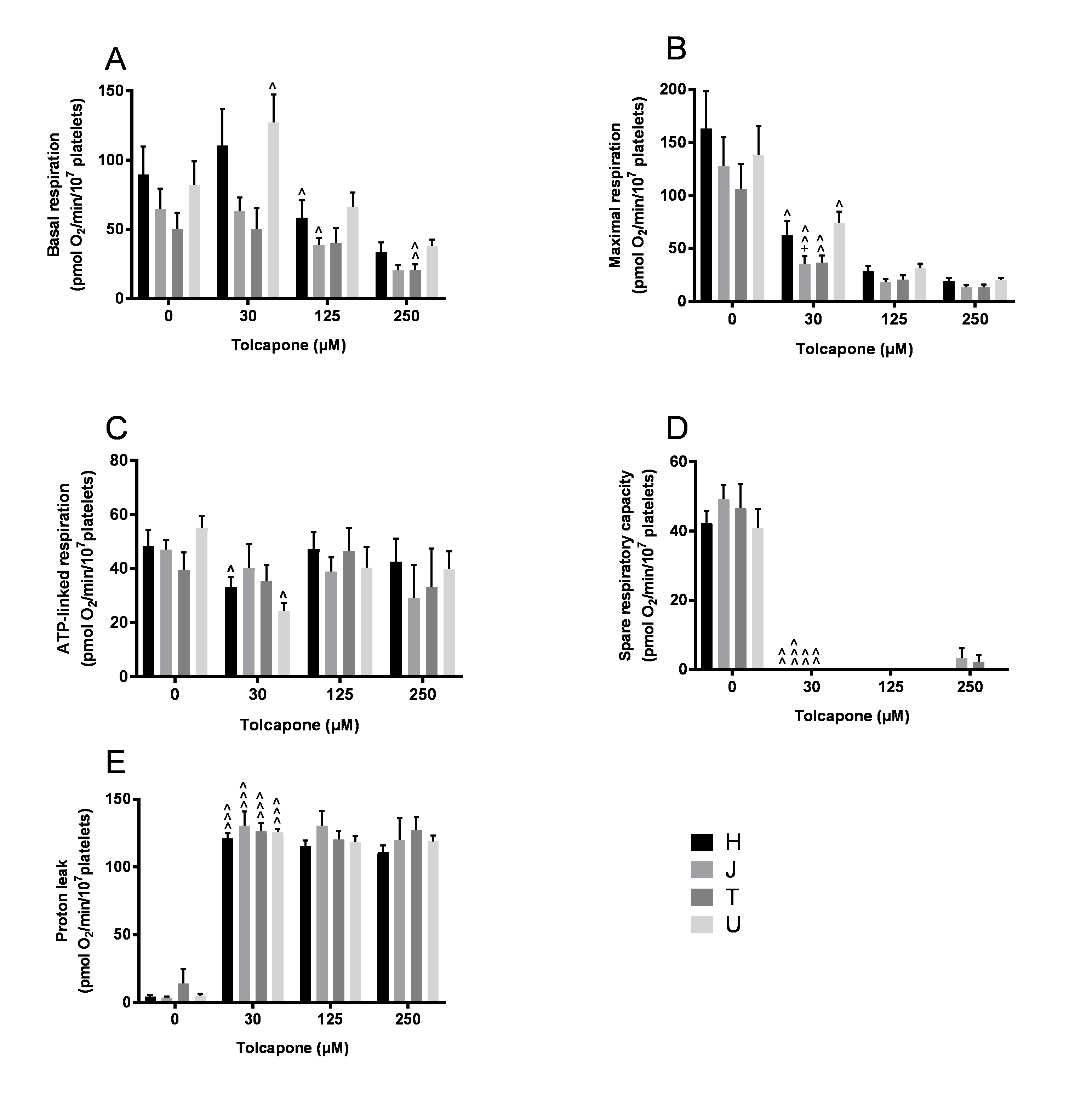

Supplement: Supplementary file 8 — Supplementary Figure 8: Mitochondrial function of tolcapone-treated platelets. Extracellular flux analysis of platelets from donors of haplogroups H, J, T and U was performed following acute treatment with tolcapone. A, B: changes in maximal and basal respiration, C-E: changes in ATP-linked respiration, spare respiratory capacity and proton leak respectively. Statistical significance compared to vehicle control: ^ p < 0.05, ^^ p < 0.01, ^^^ p < 0.001. For clarity only the first point of significance is shown. Statistical significance of haplogroups J, T compared to other haplogroups; + p < 0.05. Data are presented as mean + SEM of n ≥ 6 independent experiments (TIF 292 kb) [file 204_2021_2988_MOESM8_ESM.tif]
